# The prospective evaluation and risk factors of dysphagia after surgery in patients with oral cancer

**DOI:** 10.1186/s40463-020-00479-6

**Published:** 2021-01-25

**Authors:** Takumi Hasegawa, Nanae Yatagai, Tatsuya Furukawa, Emi Wakui, Izumi Saito, Daisuke Takeda, Yasumasa Kakei, Akiko Sakakibara, Ken-ichi Nibu, Masaya Akashi

**Affiliations:** 1 Department of Oral and Maxillofacial Surgery, Kobe University Graduate School of Medicine, 7-5-1 Kusunoki-cho, Chuo-ku, Kobe, 650-0017 Japan; 2 Department of Otolaryngology-Head and Neck Surgery, Kobe University Graduate School of Medicine, Kobe, Japan

**Keywords:** Prospective, Dysphagia, Quality of life, Oral carcinoma, Head and neck

## Abstract

**Background:**

This prospective study investigated the change of swallowing ability using the Swallowing Ability Scale System (SASS) and swallowing-related quality of life (QOL) by Performance Status Scale for Head and Neck Cancer patients (PSS-H&N). This study also investigated the risk factors for postoperative dysphagia in patients who received reconstructive surgery for oral cancer.

**Subjects and Methods:**

This study included 64 patients (33 men and 31 women) who underwent radical surgery with neck dissection and reconstructive surgery for oral cancers between July 2014 and February 2018. We evaluated risk factors for poor swallowing ability after treatment, including demographic factors, preoperative factors and perioperative factors, with univariate and multivariate analyses. The change of swallowing ability by the SASS and swallowing-related QOL by PSS-H&N were evaluated prospectively prior to the initiation of surgery within 1 week and at 1 and 3 months after treatment.

**Results:**

Advanced T stage (T3, 4) (odds ratio (OR) = 79.71), bilateral neck dissection (OR = 20.66) and the resection of unilateral or bilateral suprahyoid muscles (OR = 17.00) were associated with poor swallowing ability after treatment. The scores for time for food intake and Eating in Public were associated with decrease of QOL in the poor group.

**Conclusions:**

We propose that clinicians consider the risk factors identified in this study and pay close attention to the management of oral cancer patients with reconstructive surgery.

**Graphical abstract:**

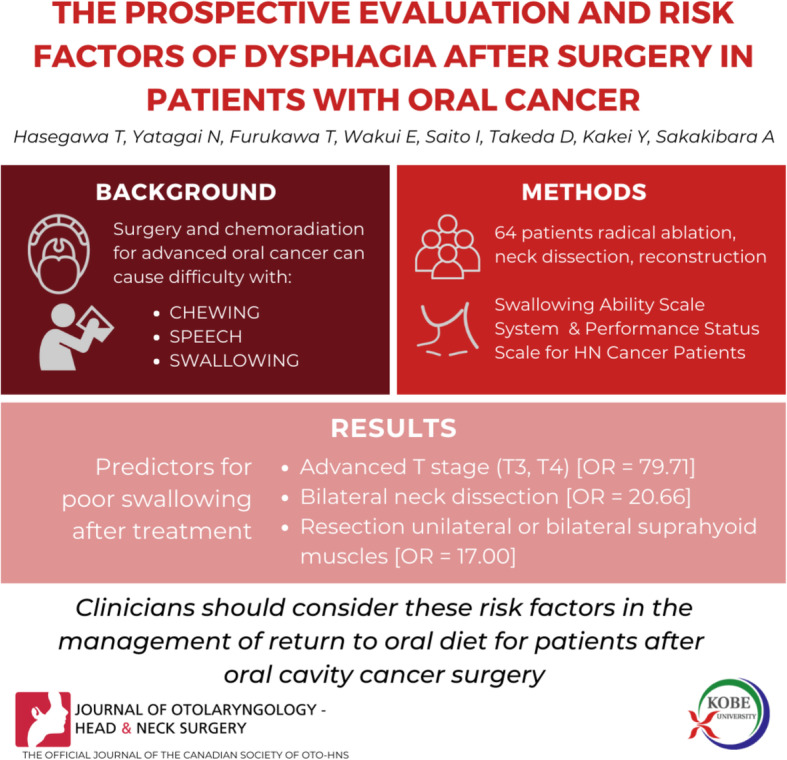

## Introduction

The treatment strategies for oral cancer have been improving and have reduced postoperative mortality and increased the survival rate of oral cancer patients [1]. Many issues around the major functional loss arising after treatment have been improved by microsurgical reconstructive techniques [2]. However, surgery and chemoradiotherapy for advanced oral cancer often cause severe disabilities, such as disfigurement and problems with chewing, speech and swallowing [3–5]. The impact of oral cancer resection and reconstruction on swallowing functions has been evaluated in several studies [6, 7]. Various risk factors of postoperative dysphagia have been identified, including poor performance status, the location of resection, anterior or extensive mandibular bone resection, method of reconstruction, tongue mobility and volume, and postoperative radiotherapy [7]. Posttreatment function and quality of life (QOL) is influenced by various factors such as T stage, N stage and neck dissection [3, 8, 9]. The deterioration of QOL by decreased postoperative function can lead to socio-economic failure, depression and, eventually, suicide [10, 11].

Numerous subjective and objective evaluation of swallowing ability measures are available [7]. The major objective evaluation for swallowing ability is videofluoroscopic evaluation (VE) [7, 12, 13]. Clinical evaluations are widely performed by various functional tests [7]. These tests that conduct subjective evaluation include grading systems such as the Swallowing Ability Scale System (SASS) [14], the M.D. Anderson Dysphagia Inventory [15], the Performance Status Scale for Head and Neck Cancer patients (PSS-H&N) [16], and the Functional Assessment of Cancer Therapy-H&N (FACT-H&N) [17]. However, some scales involved multiple questionnaire and can be too difficult to understand for older patients [15, 17]. Fujimoto et al. reported the SASS using the MTF score that can easily evaluate swallowing function [14]. The SASS is useful for bedside evaluation because the test is simple and easy and can evaluate by referring to actual feeding condition.

This prospective study investigated the change of swallowing ability using SASS and swallowing-related QOL by Performance Status Scale for Head and Neck Cancer patients (PSS-H&N). This study also investigated the risk factors for postoperative dysphagia in patients who received reconstructive surgery for oral cancer.

## Methods

This was a non-randomized prospective cohort study. This study was approved by the institutional review board of Kobe University Graduate School of Medicine and by the institutional review boards of the participating hospitals (authorization number: 1603). The patient group included 64 patients (33 men and 31 women) who underwent radical surgery with neck dissection and reconstructive surgery for oral cancers between July 2014 and February 2018 at the Department of Oral and Maxillofacial Surgery, Kobe University Hospital. The mean patient age was 66.9 ± 13.6 years (range: 15–88 years). Inclusion criteria were as follows: a histological diagnosis of oral squamous cell carcinoma, the presence of a previously untreated tumor scheduled for radical surgery with neck dissection at initial visit and the absence of other suspected distant metastasis. Patients who had undergone neoadjuvant radiotherapy (RT) or chemotherapy or with inadequate clinical information were excluded.

All subjects included in this study were assessed by an otolaryngologist and speech therapist before and after radical surgery. After VE was performed and the function of swallowing was evaluated by the otolaryngologist, patients started swallowing and speech rehabilitation programs as soon as their clinical condition allowed correct acceptance, usually 1 week after the surgical procedure. Re-evaluation of swallowing ability was performed and the decision for rehabilitation programs was discussed weekly by the attending physician, speech therapist, nurse and dietitian. Rehabilitation programs included indirect and direct trainings. Indirect trainings were oral care and active movement exercises, in which the patient protrudes and then retracts the tongue, licks the sides of both cheeks, licks the lips and rolls the tongue up to the soft palate. In addition, sensory procedures were performed to stimulate the patient’s soft palate and tongue base with a swab dipped in ice water. Active and passive jaw movement exercises were also performed. Direct trainings were the adjustment of food form and instruction of therapeutic postures and swallowing procedures such as supraglottic or alternate swallowing. Therapeutic postures and exercises were instructed to maximize the swallow function and minimize aspiration under the guidance of a therapist. For unilateral affected dysphagia patients, head rotation to the affected side was introduced to prevent aspiration. Patients were also guided to lower the chin to the chest before swallowing.

Data assessed for each patient included (1) demographic factors (sex, age, smoking history, alcohol drinking, performance status, American Society of Anesthesiologists [ASA] Physical Status Classification, body mass index and body weight on hospital discharge), (2) preoperative factors (tumor subsite, T stage, N stage, total protein [TP] and albumin [Alb]) and (3) perioperative factors (unilateral or bilateral neck dissection, radical or selective neck dissection, the types of reconstruction flap, the presence or absence of postoperative RT or chemoradiotherapy, the resection of unilateral or bilateral suprahyoid muscles, surgical site infection, blood loss, surgical time and blood transfusion). T and N stage were decided by preoperative examination, including computed tomography (CT) and magnetic resonance imaging of the head and neck region. Preoperative distant metastasis was excluded based on the findings of chest CT or PET. The data on demographic factors were collected by interview and measurement at hospital admission and discharge. The data on perioperative factors were collected by referring to intraoperative and postoperative findings. The resection of suprahyoid muscles was defined the case in which all muscles including mylohyoid, digastric and geniohyoid muscle were resected except for stylohyoid muscles. SSI was defined in accordance with the guideline issued by the Centers for Disease Control and Prevention [18]; it included purulent discharge from any incision or organ space within 30 days postoperatively, with or without microbiological evidence. Details of these characteristics and patient demographics are listed in Table 1.

**Table 1:** Characteristics of patients according to swallowing ability according to SASS

Characteristics	Swallowing ability		*P* value
	Good n (%)	Poor n (%)	
Number of patients	41 (64.1)	23 (35.9)	
Sex			
Male	20 (48.8)	13 (56.5)	0.425 α
Female	21 (51.2)	10 (43.5)	
Age			
Range (years)	24–82	15–88	
Mean ± SD	66.4 ± 12.7	67.7 ± 15.2	0.485 β
Smoking history			
No	28 (68.3)	12 (52.2)	0.282 α
Yes	13 (31.7)	11 (47.8)	
Alcohol drinking			
No	24 (58.5)	12 (52.2)	0.793 α
Yes	17 (41.5)	11 (47.8)	
Performance status			
0	36 (87.8)	17 (73.9)	0.182 α
1	5 (12.2)	6 (26.1)	
ASA Physical Status Classification			
1	7 (17.1)	5 (21.7)	0.742 α
2	34 (82.9)	18 (78.3)	
BMI			
Range (kg/m^2^)	16.0–32.1	14.5–27.2	
Mean ± SD	22.9 ± 3.9	21.1 ± 3.3	0.109 β
Body weight on hospital discharge			
Range (kg/m^2^)	32.5.0–81.0	35.2–69.3	
Mean ± SD	55.6 ± 12.4	50.2 ± 8.3	0.084 β
Total protein			
Range (g/dl)	6.1–8.1	6.4–8.1	
Mean ± SD	7.0 ± 0.5	7.2 ± 0.5	0.389 β
Albumin			
Range (g/dl)	3.0–4.8	3.3–5.0	
Mean ± SD	4.2 ± 0.5	4.1 ± 0.4	0.236 β
Subsite			
Tongue	13 (31.7)	6 (26.1)	0.878 γ
Buccal mucosa	5 (12.2)	3 (13.0)	
Floor of the mouth	3 (7.3)	2 (8.7)	
Upper gingiva	6 (14.6)	3 (13.0)	
Lower gingiva	12 (29.3)	9 (39.1)	
Other	2 (4.9)	0 (0)	
Tongue	13 (31.7)	6 (26.1)	0.250 α
Others	28 (68.3)	17 (73.9)	
T stage			
1	1 (2.4)	0 (0)	< 0.001 * γ
2	30 (73.2)	6 (26.1)	
3	3 (7.3)	9 (39.1)	
4a/b	7 (17.1)	8 (34.8)	
1, 2	31 (75.6)	6 (26.1)	< 0.001 * α
3, 4	10 (24.4)	17 (73.9)	
N stage			
0	30 (73.2)	10 (43.5)	0.057 γ
1	6 (14.6)	6 (26.1)	
2b	5 (12.2)	5 (21.7)	
2c	0	2 (8.7)	
0	30 (73.2)	10 (43.5)	0.031 * α
1, 2	11 (26.8)	13 (56.5)	
Bilateral neck dissection			
No	38 (92.7)	14 (60.9)	0.005 * α
Yes	3 (7.3)	9 (39.1)	
Type of neck dissection			
Selective neck dissection	34 (82.9)	12 (52.2)	0.019 * α
Modified radical neck dissection	7 (17.1)	11 (47.8)	
Resection of unilateral/bilateral suprahyoid muscles			
No	25 (61.0)	6 (26.1)	0.010 * α
Yes	16 (39.0)	17 (73.9)	
No	25 (61.0)	6 (26.1)	0.019 * γ
Resection of unilateral suprahyoid muscles	14 (34.1)	13 (56.5)	
Resection of bilateral suprahyoid muscles	2 (4.9)	4 (17.4)	
Types of reconstruction flap			
Forearm	29 (70.7)	11 (47.8)	0.320 γ
Rectus abdominis	6 (14.6)	7 (30.4)	
Fibular	5 (12.2)	4 (17.4)	
Pectoral major musculocutaneous	1 (2.4)	1 (4.3)	
Radial forearm	29 (70.7)	11 (47.8)	0.106 α
Others	12 (29.3)	12 (52.2)	
Blood loss			
Range (ml)	160–2358	180–2345	
Mean ± SD	683.7 ± 467.3	695.5 ± 459.6	0.845 β
Operation time			
Range (min)	345–917	517–832	
Mean ± SD	646.1 ± 120.7	706.7 ± 80.5	0.035 * β
Blood transfusion			
No	32 (78.0)	13 (56.5)	0.091 α
Yes	9 (22.0)	10 (43.5)	
Surgical site infection			
No	31 (75.6)	19 (82.6)	0.754 α
Yes	10 (24.4)	4 (17.4)	
Postoperative radiotherapy or chemoradiotherapy			
No	31 (75.6)	13 (56.5)	0.161 α
Yes	10 (24.4)	10 (43.5)	

α: Fisher’s exact test; β: Mann–Whitney U test; γ: Chi-squared test. * *P* < 0.05

The patients were classified into two groups according to SASS scores at 3 months after treatment as follows: poor (MTF score ≤ 9 points) or good (MTF score 10–15 points)

Functional swallowing evaluations were performed using SASS. The SASS was based on the MTF classification [14]: method of food intake (M), time for food intake (T) and the group of the food that can be taken (F). For each of these parameters, five subgroups are classified and scored (Table 2).

**Table 2:** The SASS was based on the MTF classification

**The method of food intake (M score)**	
M1	Tube feeding is the only method of intake
M2	Small portions of food can be eaten, but tube feeding is the main method of intake
M3	Capacity to eat anything if the food is prepared in a suitable form
M4	Almost all food can be swallowed, but care must be taken to avoid aspiration
M5	All food can be swallowed
**The average time for food intake (T score)**	
T1	Intake of food requires more than 50 min or is impossible
T2	Intake of food requires 35 to 45 min
T3	Intake of food requires 25 to 35 min
T4	Intake of food requires 15 to 25 min
T5	Normal food intake time, < 15 min
**The group of the food that can be taken (F score)**	
F1	Only no viscous fluids can be swallowed
F2	Viscous fluids can be swallowed
F3	Gruel food can be eaten
F4	Soft food such as cooked rice or vegetables can be eaten
F5	Any type of food can be eaten

The swallowing-related QOL was evaluated using PSS-H&N [16]. PSS-H&N is a clinician-rated instrument and divided to three categories: Eating in Public, Understandability of Speech and Normalcy of Diet (Table 3). For each of these parameters, subgroups were classified and scored from 0 to 100. The Eating in Public demonstrated swallowing-related QOL by documenting the patient’s ability to share a meal with others and in what type of environment. The Understandability of Speech demonstrated the degree to which the listener can understand the patient’s speech. The Normalcy of Diet subscale demonstrated the extent to which the patient can eat a regular diet. The change of swallowing ability by SASS and swallowing-related QOL by PSS-H&N were evaluated prospectively prior to the initiation of surgery within 1 week and at 1 and 3 months after treatment. We defined “after treatment” as the period of time after the completion of surgery or surgery and adjuvant therapy. In this study, the SASS scores at 3 months after treatment were decided as the primary outcome, and the changes in the SASS and PSS-H&N scores were decided as the secondary outcome. In this study, the Understandability of Speech of PSS-H&N was not used an outcome. Therefore, the results were not include in this study. To grade the results and to analyze the final outcome in relation to other clinical factors, patients were classified into two groups according to SASS scores as follows: poor (MTF score ≤ 9 points) or good (MTF score 10–15 points).

**Table 3:** Performance Status Scale for Head and Neck Cancer Patients

**Eating in Public**	
100	No restriction of place, food, or companion (eats out at any opportunity)
75	No restriction of place, but restricts diet when in public (eats anywhere, but may limit intake to less “messy” foods, e.g., liquids)
50	Eats only in presence of selected persons in selected places
25	Eats only at home in presence of selected persons
0	Always eats alone
**Understandability of Speech**	
100	Always understandable
75	Understandable most of the time; occasional repetition necessary
50	Usually understandable; face-to-face contact necessary
25	Difficult to understand
0	Never understandable; may use written communication
**Normalcy of Diet**	
100	Full diet (no restrictions)
90	Peanuts
80	All meat
70	Carrots, celery
60	Dry bread and crackers
50	Soft, chewable foods (e.g., macaroni, canned/soft fruits, cooked vegetables, fish, hamburger, small pieces of meat)
40	Soft foods requiring no chewing (e.g., mashed potatoes, apple sauce, pudding)
30	Pureed foods (in blender)
20	Warm liquids
10	Cold liquids
0	Non-oral feeding (tube fed)

All of the variables associated with the poor group were introduced into a multiple logistic regression model. For analyses of variables with more than three categorical data, patients were divided by tumor sites (tongue vs. others); T stage (T1, T2, vs. T3, T4); N stage (N0 vs. others); the types of reconstruction flap (forearm vs. others) and the resection of suprahyoid muscles (bilateral conservation vs. others).

### Statistical analysis

SPSS 22.0 (SPSS, Chicago, IL) and Ekuseru-Toukei 2012 (Social Survey Research Information Co., Ltd., Tokyo, Japan) were used for the statistical analyses. The association of each variable with the poor group was analyzed by the Mann-Whitney U nonparametric test for ordinal variables and the Fisher’s exact test or the Chi-squared test for categorical variables. Probabilities of less than 0.05 were accepted as significant. All of the variables associated with the poor group was introduced into a multiple logistic regression model. Forward stepwise algorithms were used, with the rejection of those variables that did not fit the model significantly. Odds ratio (OR) and 95% confidence intervals (CIs) were also calculated.

## Results

The number of patients in the good and poor groups, according to the SASS scores at 3 months after treatment as described in Methods, was 41 (64.1%) and 23 (35.9%) patients, respectively. In univariate analysis, advanced T stage (*P* <  0.001), advanced N stage (*P* = 0.013), bilateral neck dissection (*P* = 0.005), modified radical neck dissection (MRND) (*P* = 0.019), the resection of unilateral or bilateral suprahyoid muscles (*P* = 0.010) and longer operation time (*P* = 0.035) were significantly associated with poor swallowing ability (Table 1). No association with poor swallowing ability was observed for other studied factors. With regard to the operative factors according to T stage, there was no significant difference between advanced T stage and extensive neck dissection, extensive resection of suprahyoid muscle, and adjacent organs. However, there were many cases of resection of mandible among cases with advanced T stage (*P* = 0.021) (Table 4).

**Table 4:** Operative factors according to T stage

Characteristics	T stage		*P* value
	1, 2 n (%)	3, 4 n (%)	
Number of patients	37 (57.8)	27 (42.2)	
The resection of mandible			
No	21 (56.8)	7 (25.9)	0.021 * α
Yes	16 (43.2)	20 (74.0)	
The resection of adjacent organ			
No	29 (78.4)	17 (63.0)	0.260 α
Yes	8 (21.6)	10 (37.0)	
Bilateral neck dissection			
No	30 (81.1)	22 (81.5)	1.000 α
Yes	7 (18.9)	5 (18.5)	
Resection of unilateral/bilateral suprahyoid muscles			
No	17 (45.9)	14 (51.9)	0.801 α
Yes	20 (54.1)	13 (48.1)	

α: Fisher’s exact test. * *P* < 0.05

A logistic regression model with forward stepwise algorithms showed that advanced T stage (T3, 4) (OR = 79.71) (*P* = 0.001), bilateral neck dissection (OR = 20.66) (*P* = 0.010) and the resection of unilateral or bilateral suprahyoid muscles (OR = 17.00) (*P* = 0.012) were significantly associated with poor swallowing ability (Table 5).

**Table 5:** Results of multivariate logistic regression analysis of the risk factors for poor swallowing ability

			95% CI	
Variable	*P* value	Odds ratio	Lower	Upper
T stage (T3, 4)	0.001	79.71	6.67	952.60
Bilateral neck dissection	0.010	20.66	2.06	206.97
Resection of unilateral or bilateral suprahyoid muscles	0.012	17.00	1.84	156.70

*CI* Confidence interval

**Fig. 1:**
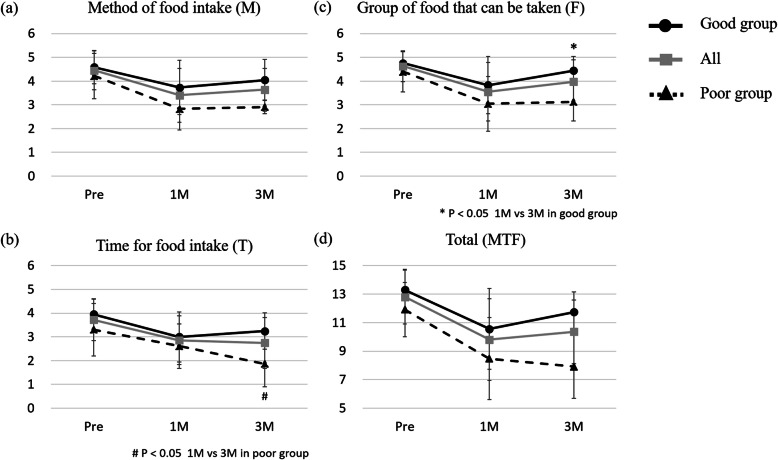
The change of swallowing ability by Swallowing Ability Scale System. (**a**) Method of food intake (M), (**b**) time for food intake (T), (**c**) group of food that can be taken (F), and (**d**) total (MTF)

The scores of all groups at 3 months after treatment were significantly lower than the scores before surgery (*P* <  0.05) (Fig. 1a, b, c, d). In the poor group, the T score at 3 months after treatment was significantly decreased from that at 1 month after treatment (*P* = 0.013) (Fig. 1b). Among the poor, good and overall groups, the M and F scores at 3 months after treatment were higher than those at 1 month. In the good group, the F scores at 3 months after treatment were significantly higher than those at 1 month after treatment (*P* = 0.022) (Fig. 1c). Analysis of the total MTF scores showed that the score of the good group at 3 months after treatment was higher than at 1 month (Fig. 1d). In the poor group, the total MTF scores at 3 months after treatment decreased compared with scores at 1 month. However, there were no significant differences.

**Fig. 2:**
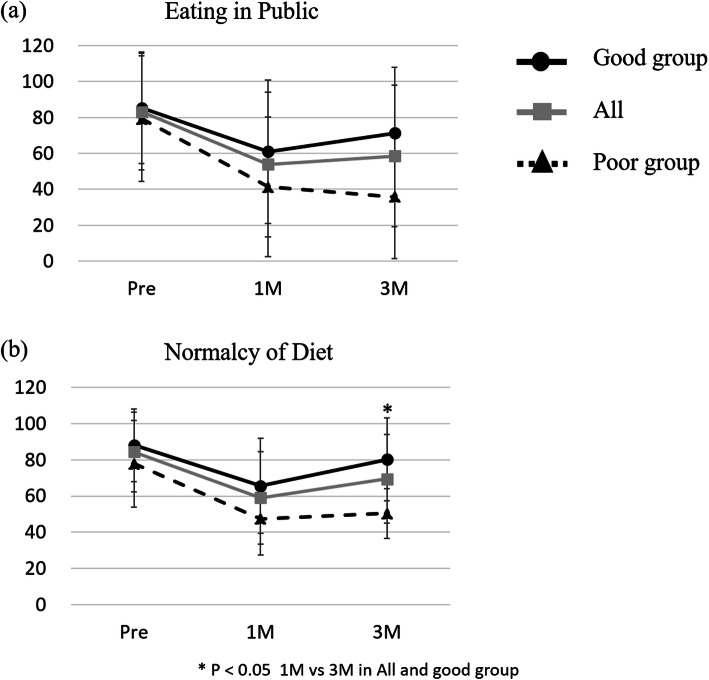
The change of swallowing-related QOL by the Performance Status Scale for Head and Neck Cancer patients. (**a**) Eating in Public, (**b**) Normalcy of Diet

Regarding PSS-H&N score, the scores of the good group at 3 months after treatment were significantly higher than the scores of the poor group at 3 months after treatment (*P* <  0.05) (Fig. 2a, b). The scores of all groups at 3 months after treatment were significantly lower than the scores before surgery (*P* <  0.05). Regarding Normalcy of Diet scores for swallowing-related QOL, all groups showed higher scores at 3 months after treatment than at 1 month (Fig. 2b). In overall patients group and good group showed significantly higher scores at 3 months after treatment than at 1 month (*P* = 0.016, *P* = 0.006) (Fig. 2b). The poor group showed a decreased Eating in Public score at 3 months after treatment compared with at 1 month (Fig. 2a). However, there was no significant difference.

## Discussion

Oral and oropharyngeal cancer patients are reported to suffer a higher risk of posttreatment dysphagia with less than half oral intake achieved compared with patients with cancers in other sites of the head and neck [19]. The postoperative swallowing ability can be influenced by many factors, including additional treatments such as RT and chemoradiotherapy, operative factors and patient-related factors such as wound healing, rehabilitation and personal motivation [20]. Early posttreatment dysphagia is mainly related to reduced tongue base retraction and laryngeal elevation. In contrast, late posttreatment dysphagia is related to delayed pharyngeal swallowing and incomplete cricopharyngeal opening [21]. In particular, suprahyoid muscles play important roles in hyoid and laryngeal elevation and are related to early posttreatment dysphagia. Laryngeal penetration and aspiration are caused by poor hyoid/laryngeal elevation and poor opening of the entry into esophagus. The suprahyoid muscles are involved with depression of the mandible and subsequent opening of the mouth, movement of the tongue as secondary muscles of mastication. Surgical resection of tumors can damage structures, such as the muscles that control swallowing. Furthermore, extensive surgery and RT can lead to tissue fibrosis and edema [22–24]. In this study, advanced T stage (T3, 4) (OR = 79.71) (*P* = 0.001) and the resection of unilateral or bilateral suprahyoid muscles (OR = 17.00) (*P* = 0.012) were significantly associated with poor swallowing ability. These results of T stage were consistent with other reports [8, 9, 25]. In the analyses of operative factors according to T stage, there was no significant difference between advanced T stage and extensive neck dissection, extensive resection of suprahyoid muscle, and adjacent organs. Instead, there were many cases of resection of mandible in cases with advance T stage (*P* = 0.021). Therefore, tooth loss, trismus, and extensive resection of suprahyoid muscle with resection of mandible may affect postoperative dysphagia. The reconstruction of bone structures and occlusion such as dental implant may thus be useful. In contrast, postoperative RT or chemoradiotherapy was not significantly associated with poor swallowing ability in this study. However, several investigators previously described an effect of RT on poor swallowing ability [5, 20]. The difference of results may be due to short-term observation (3 months) in this study. Postoperative RT or chemoradiotherapy may thus influence the swallowing ability at 6 months or 1 year by the progression of tissue fibrosis. Future research should investigate swallowing ability over the long term.

In a study of dysphagia in tongue cancer patients, Son et al. reported that patients with N1 or N2 stage had a higher incidence of aspiration than N0 stage patients. The authors also reported that patients who underwent MRND had a higher incidence of aspiration than those who underwent supraomohyoid neck dissection [9]. N2 stage and radical neck dissection are correlated with a more advanced disease and more extensive resection. As result, dysphagia and aspiration are triggered. In this study, MRND in univariate analysis and bilateral neck dissection (OR = 20.66) (*P* = 0.010) in multivariate analysis were significantly associated with poor swallowing ability. One reason may be the disturbance of laryngeal elevation by neck dissection and the resection of suprahyoid muscles. Therefore, we try to preserve suprahyoid muscles intraoperatively without increasing the risk of recurrence. In addition, we performed surgery such as laryngeal suspension to improve swallowing function for high risk patients with postoperative dysphagia (the resection of bilateral suprahyoid muscles). In case of bilateral neck dissection and the resection of bilateral suprahyoid muscles, decrease of swallowing ability cannot be completely prevented, although laryngeal suspension has a certain effect to swallowing ability.

Generally, the superiority of fasciocutaneous flap reconstruction such as forearm flap provides satisfying replacement of oral structures without disturbance to the mobility of the floor of the mouth and tongue elevators compared with bulky myocutaneous flap reconstruction [26–28]. However, Kalavrezos et al. demonstrated that the use of composite flaps has no adverse impact on swallowing recovery [20]. Similarly, in this study, the type of reconstruction flap was not significantly associated with poor swallowing ability.

The treatment of oral cancer inhibits this social function causing marked deterioration in QOL [29]. In a study of patients with primary resection of tongue cancer and free flap reconstruction, most of the patients had postoperative dysphagia including difficulty with swallowing liquids in the early postoperative phase [7]. Rieger et al. also reported that swallowing ability with liquid showed the largest decrease at the early postoperative time and then increased at 6 months postoperatively [30]. In this study, in the good group, the F scores at 3 months after treatment were significantly higher than those at 1 month after treatment (*P* = 0.022). Regarding the Normalcy of Diet of PSS-H&N score, the overall patient group and good group showed significantly higher scores at 3 months after treatment than at 1 month (*P* = 0.016, *P* = 0.006, respectively). These results suggested that postoperative swallowing ability decreased the most at the early postoperative phase (1 month postoperatively) and then increased, similar to previous studies [7, 30]. In contrast, in the poor group of this study, the T score at 3 months after treatment was significantly decreased compared with 1 month after treatment (*P* = 0.013). These results suggest that if patients with risk factors are managed more intensively between 1 month and 3 months after treatment, postoperative dysphagia at 3 months after treatment may possibly be improved in the poor group. In this study, the scores for time for food intake and Eating in Public were associated with decrease of QOL (Eating in Public) in the poor group. The patients in the poor group might have challenges to adjust the form of food intake because of insufficient support from the social environment such as from the medical staff or the patients’ family members after discharge from a hospital. Thus, psychological factors caused by longer time for food intake may negatively impact eating in public. However, these findings and speculation should be carefully considered because of many various confounding factors and the small sample size.

This study had several limitations. First, the present prospective study was nonrandomized and patients were relatively heterogeneous regarding the defect. In addition, QOL is associated many other factors such as social interaction and psychosocial factors (e.g., anxiety, depression) other than the evaluated factors in this study. Therefore, although multivariate analysis was performed to decrease the effect of confounding factors as much as possible, bias could not be completely excluded. Second, this study evaluated function within 3 months after treatment, which may not reflect ultimate swallowing ability and swallowing-related QOL. Therefore, the timeframe might be too short to quantify swallow recovery. Third, we did not use objective evaluations such as VE that might have provided a more sensitive measure of the risk factors on posttreatment dysphagia. Also, the sample size might be inadequate to analyze the subjective data of swallowing ability and swallowing-related QOL. Future research should involve a large-scale cohort study over the long-term and investigate predictors of dysphagia including objective swallowing evaluations at these same time points.

In conclusion, we successfully demonstrated the change of swallowing ability by SASS and swallowing-related QOL by PSS-H&N and the risk factors for postoperative dysphagia in patients who received reconstructive surgery for oral cancer. The scores for time for food intake and Eating in Public were associated with decrease of QOL in the poor group. Advanced T stage (T3, 4), bilateral neck dissection and the resection of unilateral or bilateral suprahyoid muscles were significantly associated with poor swallowing ability. We propose that clinicians consider these risk factors and pay close attention to the management of oral cancer patients with reconstructive surgery. Suprahyoid muscles may have to be preserved intraoperatively as much as possible, if the risk of recurrence do not increases.

## Data Availability

The datasets of the current study are available from the corresponding author on reasonable request.
